# Quantifying Components in a Model Vaccine with Machine-Learning-Augmented Raman Spectroscopy

**DOI:** 10.1021/acs.analchem.5c05538

**Published:** 2026-04-28

**Authors:** Jana Hahn, Pooja Gune, Sascha Hein, Wolf Holtkamp, Marcel H. Schulz, Walter Matheis, Volker Öppling, Christel Kamp

**Affiliations:** † Allergology Division, Central Method Development Section, 39053 Paul-Ehrlich-Institut, 63225 Langen, Germany; ‡ Bioinformatics, Goethe University Frankfurt, 60325 Frankfurt am Main, Germany; § Institute for Computational Genomic Medicine, Goethe University Frankfurt, 60590 Frankfurt am Main, Germany; ∥ Infectious Diseases Division, Quality Assessment Vaccines Section, Paul-Ehrlich-Institut, 63225 Langen, Germany; ⊥ Infectious Diseases Division, Product Testing Vaccines Section, Paul-Ehrlich-Institut, 63225 Langen, Germany

## Abstract

Vaccination is a highly efficient strategy in controlling infections. Aluminum-containing adjuvants have long been used to enhance immunogenicity, but quantification of adsorbed antigens remains analytically challenging. This study uses Raman spectroscopy, a powerful, nondestructive technique, augmented by machine learning to characterize model vaccines comprising Bovine Serum Albumin adsorbed to aluminum hydroxide. Spectral fingerprints of the pure components, their contributions to the vaccine mixtures, and the resulting concentrations were estimated using autoencoder and benchmarked against Multivariate Curve Resolution (MCR), a state-of-the-art linear deconvolution method. We implement a custom autoencoder (AE) architecture featuring a scale-insensitive reconstruction loss and a concentration-anchored latent space, which we compare against Multivariate Curve Resolution (MCR) to evaluate linear versus nonlinear decomposition efficacy. By integrating synthetic spectra based on the Contextual Out-of-Distribution Integration (CODI) method, we achieved a concentration prediction accuracy that aligns with standards for biological reference methods. While MCR proved more robust for recovery of component spectra, the AE demonstrated superior performance in estimating concentrations. These results highlight the potential for characterization of adsorbed vaccines and offer a pathway for improved quality control in biopharmaceutical formulations.

## Graphical abstract

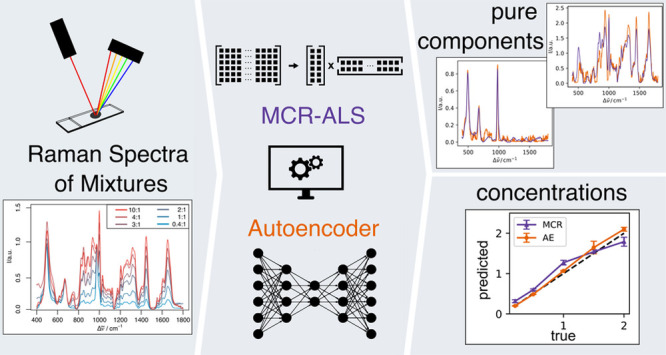

## Introduction

Raman spectroscopy, a nondestructive, label-free method, has shown great potential for quality and process control in the pharmaceutical industry.^1,2^ While early applications have largely focused on small molecule drugs, the role of Raman spectroscopy in characterizing biological samples^3^ and biomedicines has gained increasing attention over the years.^4–6^ A major challenge for Raman spectroscopic analyses of these samples is their inherent variability and the often low intensity of protein spectra of interest compared to excipient spectra.^4,7^ This holds particularly for vaccines, which typically contain proteins as active pharmaceutical ingredient (API) in aqueous solution within a matrix of excipients. Yet, any improvement in the mandatory quality control of vaccines has immediate impact on public health given that vaccination is a major and highly efficient strategy in controlling infections.

The enhancement of vaccine immunogenicity by adjuvants has been instrumental and a wide range of vaccines use aluminum-based adjuvants, such as aluminum hydroxide (Al­(OH)_3_).^8,9^ Within these vaccine formulations, proteins are typically adsorbed to crystalline structures of aluminum salts, which poses significant challenges to analytical methods for vaccine drug product characterization, as compendial methods require desorption of the antigens prior to identification.^10^ Regulatory authorities often require testing of identity and potency in the final product formulation. Potency testing is typically done in animal experiments.^11^ Consistency approaches aim for a reduction of animal experiments, i.e., by replacement of animal based potency testing with determination of antigen content and require reliable methods of protein quantification. Quantification of adsorbed antigens with classical protein assays for example using the Kjeldahl method have limitations as they do not allow for a differential analysis of antigens in multivalent vaccines.^12^ Regulatory requirements specify that quality testing should occur as late as possible in the production cycle, ideally on the final product.^11^ Consequently, methods must be developed that enable antigen quantification on the finished product. Several procedures using immunological methods were developed to characterize adsorbed antigens in final vaccines.^13–15^ However, these methods are labor intensive, require extensive development and establishment work and are not yet part of quality control procedures as regulated by the European Pharmacopoeia.

In this study, we explored the application of Raman spectroscopy combined with computational spectral deconvolution methods for vaccine characterization. The approach aims to identify the spectral contribution of vaccine components within the spectrum of the full product and to determine their concentration ratios, enabling conclusions about the concentration of the API in the mixture. Several research groups have addressed the challenge of spectral unmixing and concentration estimation in mixtures, particularly in hyperspectral imaging and pharmaceutical analysis.^16–18^ Many of these approaches are based on linear mixing models, which may fail to accurately represent systems involving adsorbed proteins due to nonlinear interactions and saturation effects.^19^ Additionally, existing methods often depend on extensive data sets or are tailored to a single task, either unmixing or concentration prediction. One powerful computational technique that can handle small data sets for resolving mixed spectral signals and identifying individual components with their respective concentration ratios is Multivariate Curve Resolution Alternating Least Squares (MCR).^20,21^ This method operates on the basis of the underlying concept of a linear decomposition, assuming a direct relationship between the concentrations of components and the measured spectra. Another approach is to use autoencoders (AE), a neural network that has two parts, an encoder and a decoder, which are trained to represent the spectra by minimizing the reconstruction error.^16,22^ Unlike MCR, autoencoders offer more flexibility in modeling of complex relationships within the data, as they can be designed to capture various linear and nonlinear mixing models without the strict assumption of a linear decomposition. Recent frameworks, such as the dual neural network approach by Liu et al.,^23^ have integrated spectral extraction with data augmentation. Our work differentiates itself by employing a semisupervised autoencoder with a dual-constraint loss that anchors the latent space to physical concentrations. This eliminates the need for pure reference spectra, which are often unavailable or unrepresentative due to conformational changes and protein instability in the nonadsorbed state.

To benchmark the application of Raman spectroscopy combined with computational methods as a proof of concept for vaccine characterization, the adjuvant Al­(OH)_3_ and Bovine Serum Albumin (BSA), a surrogate for the active pharmaceutical ingredient, were used as a model system. Mixtures with varying concentrations of BSA adsorbed to Al­(OH)_3_ were analyzed. The therapeutic efficacy of vaccines is fundamentally determined by its identity (the correct active substance) and its content (the precise dosage). Accurate identification and quantification are essential to ensure consistent clinical performance and patient safety. The results of this application-driven proof-of-concept study demonstrate that Raman spectroscopy, combined with machine learning methods, has the potential to be applied in the quality control of vaccine formulations.

## Materials and Methods

### Experimental Protocol

#### Raman Spectroscopy

All Raman measurements were conducted using a BioRam2 (CellTool/microphotonX GmbH) Raman spectrometer. Each sample measurement contained 100 spectra with a single accumulation at 30 s exposure time. The sample area was recorded in a rectangular grid pattern to capture spatial variation and was focused with 40x microscopic objective lens with an objective correction Ring of 1.0 mm, with a laser excitation wavelength of 785 nm. The back scattered Raman light was recorded with a charge-coupled device (CCD) camera. To check the reproducibility of the spectra, each sample was measured in duplicate.

#### Sample Preparation

In the context of this study, BSA­(Merck, purity: >97%) was used as a model protein adsorbed to aluminum hydroxide (InvivoGen).^24^ We used a stock solution of 50 mg/mL BSA [0.5 g in 10 mL water] and further diluted it with water to a protein concentration of 10, 4, 3, 2, 1, 0.4 mg/mL of BSA. Adsorption was performed by mixing each BSA solution with 1 mg/mL of aluminum hydroxide adjuvant suspension [in water] at equal volumes. The resulting concentration of aluminum hydroxide matches concentration ranges found in adsorbed vaccines.^25^ The suspension was then subjected to end over end rotation [45 min] at room temperature to promote adsorption, followed by centrifugation [14,000 rpm for 5 min at room temperature] to separate aluminum adsorbed BSA (pellet) from free protein (supernatant). In one test set, the pellet was subjected to an additional washing steps [3x with 167 μL water] to remove all residual unbound protein. The presence of BSA in the pellet as well as the supernatants was confirmed by SDS-PAGE (Figure S1). Each pellet was dissolved in 500 μL, which affects the concentration of both compounds but does not affect the relative concentration ratios. In addition, aluminum adsorbed BSA was measured on CaF_2_ (Raman grade from Korth Kritalle) substrate using drop coating deposition technique.^26^

### Data Sets

The Raman measurements were divided into a training set and two independent test sets. Each set contained five distinct concentrations of BSA (0.2, 0.5, 1, 1.5, and 2 mg/mL) adsorbed to Al­(OH)_3_ (0.5 mg/mL) consistently mixed at equal volumes. These five mixtures were analyzed in duplicate, with approximately 100 Raman spectra recorded for each measurement. The duplicates measured in April 2024 were designated as the training set, while a second set measured in August 2024 served as the first test set. For the second test set, the experimental protocol was adjusted by adding a washing step to the pellet. An additional higher BSA concentration of 5 mg/mL adsorbed to 0.5 mg/mL Al­(OH)_3_ was used for testing at various points in this paper but was not included in the model training. Furthermore, a third test set was prepared for supplementary analysis by fixing the BSA concentration at 0.5 mg/mL while varying the Al­(OH)_3_ concentration (0.25, 0.34, 0.5, 1, and 2.5 mg/mL) in technical duplicates.

Furthermore, reference spectra of pure BSA (stock solution at 50 mg/mL) and pure Al­(OH)_3_ (1 mg/mL) were measured. For both, 100 spectra were recorded across the sample area, and the average spectrum was used as the reference.

### Spectral Preprocessing

The recorded Raman spectra underwent multiple preprocessing steps to ensure data quality and consistency. First, the spectral wavenumber range was restricted to 400–1800 cm^–1^, focusing on the region that contains the most relevant peaks. Cosmic spike removal was performed by identifying intensity outliers within the 100 spectra measured per sample. Outliers were defined as values that exceeded a threshold of four standard deviations and were subsequently removed from the data set. Baseline correction was achieved using a polynomial fitting approach of order four, which identified and subtracted the baseline from the spectra, resulting in a flattened spectral baseline and enhanced peak visibility. Normalization was applied to account for variations in spectral intensity. Given the fixed Al­(OH)_3_ concentration in all samples, the Raman peak at 976 cm^–1^, corresponding to Al­(OH)_3_, was used as the reference for normalization, with each spectrum scaled to the intensity of this peak to ensure comparability across samples. Finally, any negative spectral values resulting from baseline correction were set to zero to prevent inaccuracies in subsequent analyzes. The HyperSpec package was used for spectral preprocessing.^27^

### Spectral Unmixing Methods

#### Non-Negative Least-Squares Regression

Non-negative least-squares (nnls) regression is a numerical optimization technique that can be used to solve linear systems of equations with non-negativity constraints. In other words, it can find the best-fitting linear combination of a set of basis vectors that approximates a given target vector, while ensuring that all the coefficients are non-negative. In this work, the active set method by Lawson and Hanson was used.^28^ nnls was applied to find non-negative coefficients *c*_1_ and *c*_2_ that best approximate the mixture spectra as a linear combination of the pure component spectra, which are assumed to be known beforehand.

$$\min_{\begin{array}{l} c_{1},c_{2} \end{array}}\overset{b}{\underset{i=1}{\sum }}{\left(mixture\left[i\right]-\left(c_{1}\times Al{\left(OH\right)}_{3}\left[i\right]\right)+\left(c_{2}\times BSA\left[i\right]\right)\right)}^{2}$$ 1

with b being the number of spectral intensities in each spectrum. In this study, the ‘nnls’ package in R (4.2.0)^29^ was used.

#### Multivariate Curve Resolution-Alternating Least Squares

Multivariate Curve Resolution-Alternating Least Squares (MCR) proposed by Tauler^30^ is a chemometric technique that iteratively decomposes complex spectral data into its constituent pure component spectra and their corresponding concentration profiles using an alternating least-squares approach.^20^ It assumes a bilinear relationship among the experimental data, the concentrations, and the pure spectra of the components. During the iterative process, a non-negativity constraint is applied to the coefficient and spectral profiles to ensure physically meaningful results. This process continues until convergence, providing estimates of both the pure component spectra and their corresponding concentrations in the original mixtures. The estimated coefficients were linearly scaled using the training set. A single scaling factor was obtained by comparing the predicted MCR ratios with the known concentrations and then applied to all samples to yield calibrated concentration values. In this work, the MCR function from mdatools^31^ version 0.14.2 was applied using the default solver (FC-NNLS) and random matrix initialization. The number of components was set to two, corresponding to the expected number of endmembers in the mixtures.

#### Autoencoder

The convolutional autoencoder architecture was created based on the research of Georgiev et al.^22^ The encoder consists of two convolutional blocks (each 16 filters of size 3 and 5 respectively; ReLU activation; input padded with zeroes) followed by two fully connected dense layers (Leaky ReLU activation with a slope of 0.02) which projects the spectral dimension in the first dense layer down to 128 and in the second layer down to the latent space. The dimension of the latent space is equal to the number of endmembers to extract. The Decoder consists of a dense layer with a linear activation function and non-negativity kernel constraint, which projects the dimension back to the original input dimension. The autoencoder was trained using the Adam optimizer with a learning rate of 0.001 to adjust the network’s weights during training. (The training objective was to minimize a multiobjective loss function *L*_*total*_ = *SAD* + *w*_*c*_ × *MSE*. While the reconstruction fidelity was evaluated using the Spectral Angle Distance (SAD), a metric employed by Georgiev et al., we introduced an additional Mean Squared Error (MSE) term to anchor the latent activations to known concentrations. This novel dual-objective constraint forces the latent space to align with physical component abundances, with the weighting factor *w*_*c*_ set to 40 to provide an optimal trade-off between spectral endmember identification and concentration prediction accuracy. The training process consisted of maximum 10 epochs (early stopping with patience 3 and min_delta = 0.0005), each representing a complete pass through the data set, with a batch size of 8, specifying the number of samples used to update the model’s parameters in each iteration. For building the autoencoder models, TensorFlow version 2.13.0 was used.^32^

### Contextual Out-of-Distribution Integration (CODI)

Contextual out-of-distribution integration (CODI) is a technique to generate synthetic data that capture underrepresented data variation.^33^ CODI evaluates the distributional characteristics of experimental data from calibration data sets. It generates synthetic spectra *Y* around a seed spectrum *s*_*i*_ by introducing variability with independent functions *f*_1_, *f*_2_, ..., *f*_*m*_, where each function is derived from a different calibration data set {*u*_*kj*_} (eq 2).

2 $$Y=s_{i}+f_{1}+f_{2}+\cdot \cdot \cdot +f_{m}$$

Each variability function captures a specific source of variation (eq 3).

3 $$f_{j}=\overset{l_{j}}{\underset{k}{\sum }}\beta \left(0,\frac{1}{\sqrt{l_{j}}}\right)\left(u_{kj}-{\mathrm{u̅}}_{j}\right)$$

One form of such a variability function, *f*_*j*_, *j* ∈ {1, ..., *m*} can be defined as a scaled sum of the mean-centered differences from a calibration data set $\left(u_{kj}-{\mathrm{u̅}}_{j}\right)$ , where ${\mathrm{u̅}}_{j}$ is the mean spectrum of the calibration data set *j* and *u*_*kj*_ are the individual spectra within that data set. The calibration data set contains *l*_*j*_ spectra in total. The scaling factors are drawn from a Gaussian distribution with a mean of 0 and a standard deviation of $\frac{1}{\sqrt{l_{j}}}$ . These factors are applied to introduce controlled variability, ensuring the synthetic spectra reflect realistic variations observed in experimental data.

In the context of this research, we created various sets of synthetic data, each based on a different calibration set representing different sources of variability:

1. Within-Sample Variability: Variations in spectra recorded from the same sample across a rectangular grid area due to instrument noise or local inhomogeneities. Each sample measurement consists of approximately 100 Raman spectra from the same dried spot. The calibration set contains five randomly sampled mean-centered spectra per dried spot.
2. Within-Concentration ratio variability between samples of the same concentration: Variations arising from measuring the same concentration in technical duplicates (spot 1 vs spot 2) from the same pellet preparation, caused by environmental factors, slight changes in experimental setup, or temporal drifts. The calibration set contains five randomly sampled spectra per sample that are mean-centered around the concentration mean.
3. Between-concentration variability: Variations between spectra recorded from samples with different concentration ratios. Here, the mean spectrum was computed for each sample, as well as the mean spectrum across all samples. To mean-center each sample’s mean spectrum, the overall mean spectrum was subtracted from it.
4. Variability of daily polystyrene measurements: Variations in polystyrene samples used for calibration purposes of the Raman spectrometer. The calibration set contains five randomly sampled mean-centered polystyrene spectra from each of the four different measurement days. To mean-center the five sampled spectra from each measurement, the overall mean spectrum of calibration measurements was subtracted from it and represent the calibration set.
5. No added synthetic spectra.

Each spectrum in the training set was used as a seed spectrum to generate 15 synthetic spectra from the distributions found in the calibration data sets. This increases the number of spectra in the training set from approximately 1000 to 15,000. To ensure the robustness of the model and the reproducibility of the stochastic sampling, this generation process was repeated five times. Each iteration utilized a different fixed random seed (3, 15, 22, 34, and 42), which governed the random selection of the scaling factors β applied to the variability functions. All synthetic spectra were generated with the pycodi package (version 0.0.1) available via PyPI.^33^

### Metrics

For each endmember the cosine distance was computed between the predicted endmembers mi^ and the ground truth *m*_*i*_ obtained from reference measurements of the pure components (eq 4). For each endmember $m_{i}\in R_{+}^{b}$ with *b* being the number of measured spectral intensities, the cosine distance is defined as

4 cosinedistance=DC(mi,mi^)=1−mi·mi^∥mi∥∥mi^∥

with · being the scalar product and ∥∥ the Euclidean norm. The mean squared error (MSE) between the predicted ck^ and true *c*_*k*_ concentrations of BSA were evaluated (eq 5). The squared error was evaluated for each of the n individual spectra in the training or test set and then averaged across each sample spot. The values plotted in Figure 8 were further averaged across all replicates with the same concentration, and the error bars represent the variation among replicates at each concentration level.

5 MSE=1n∑k=1n(ck−ck^)2

The inverse coefficient of variation (ICV) was computed between five predicted endmembers of the same component that were predicted based on different set of additional synthetic data sets of the same size (eq 6). For each wavenumber the ICV was computed as follows: the mean signal intensity at this wavenumber *mean*(*i*_*wn*_) was divided by its standard deviation SD­(*i*_*wn*_) where *i*_*wn*_ is the set of five intensity values all measured at same wavenumber­(wn).

6 $$ICV=\frac{mean\left(i_{wn}\right)}{\mathrm{SD}\left(i_{wn}\right)}$$

## Results

After a brief introduction of the model vaccine’s design and its Raman spectral fingerprint, we identify the protein signature in the respective Raman spectra. In detail, analyses and standardization of experimental and computational procedures allow for identification and quantification of the protein component based on the full product’s Raman spectral fingerprint.

### Raman Spectral Fingerprints of Model Vaccines with Varying Protein Concentrations

Raman spectra of samples with different concentrations of Bovine Serum Albumin adsorbed to aluminum hydroxide were measured to assess the impact of protein levels on the Raman spectral fingerprint. Al­(OH)_3_ adsorbed samples were generated with a fixed concentration of Al­(OH)_3_ (0.5 mg/mL) and six different concentrations of BSA (0.2, 0.5, 1, 1.5, 2, and 5 mg/mL) in the final mixture. To confirm adsorption, pellets and supernatants were analyzed via SDS-PAGE (Figure S1). BSA was present in all pellet fractions, with residual BSA in the supernatant from samples with 2 and 5 mg/mL BSA, indicating saturation at these levels. Pellet fractions were dried on CaF_2_ slides for Raman measurements. Figure 1A shows the mixtures, alongside the reference spectra of pure Al­(OH)_3_ (blue) and pure BSA (red) (Figure 1B). Spectral features vary with the protein concentration relative to the fixed Al­(OH)_3_ concentration. Since the adjuvant concentration is known, the relative BSA concentration in each mixture can be derived from the spectral data.

**Figure 1:**
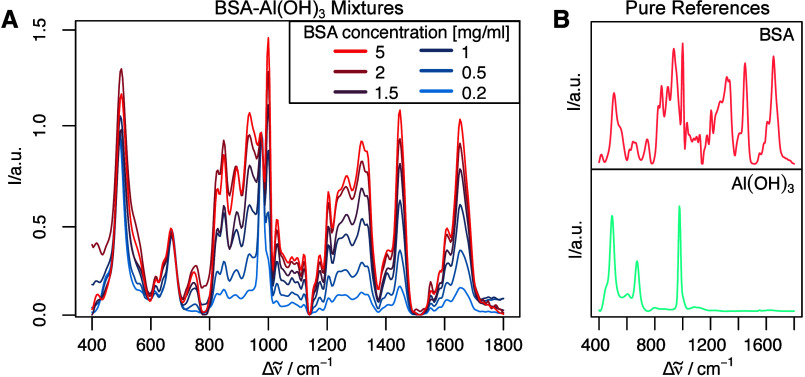
Raman spectra with varying concentration ratios of BSA adsorbed to Al­(OH)_3_ and the pure references. (A) Mixtures at six ratios (BSA concentrations in the mixture: 0.2, 0.5, 1, 1.5, 2, and 5 mg/mL adsorbed to 0.5 mg/mL Al­(OH)_3_). The gradient from blue to red in the legend indicates increasing BSA concentration, with higher concentration resulting in more red spectra. (B) Reference Raman spectra of pure Al­(OH)_3_ (blue), pure BSA (red). Each plotted spectrum is the mean of 100 spectra recorded across a single sample replicate. Spectra were measured and processed as described in the Materials and Methods Section.

### Control of Spatial Sample Heterogeneity

Drop coating deposition of samples allows for Raman measurements on dried spots, where water evaporates, leading to an increased concentration of components. This drying process enhances the spectral signal but also introduces spatial heterogeneity in the sample, which must be carefully considered in order to reliably reconstruct concentration features of the original sample. Raman spectra collected from the dried spots of BSA-Al­(OH)_3_ mixtures revealed spatial variation in spectral intensities (Figure 2) being dependent on the specific position in the sample spot. The spatial intensity pattern is influenced by the relative concentrations of protein and Al­(OH)_3_. A higher protein to Al­(OH)_3_ ratio leads to an increase in mean spectral intensity in the outer regions of the dried spots, indicative of the coffee ring effect. Protein dominant solutions tend to form a coffee ring during drying process, which occurs due to outward capillary flow transporting suspended molecules to the edges.^34^ In contrast, the samples with a lower BSA concentration do not exhibit the coffee ring effect, as the gel-like properties of Al­(OH)_3_ dominate and promote central accumulation rather than outward transport. A colormap of mean spectral intensities across dried spots of pure BSA and pure Al­(OH)_3_ can be found in Figure S2.

**Figure 2:**
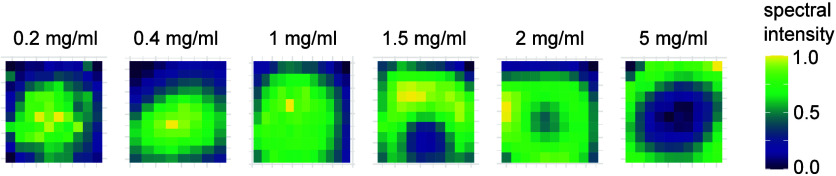
Variation in spectral intensities across dried sample spots. Intensity patterns in measured dried spots containing BSA at different BSA concentrations adsorbed to a fixed Al­(OH)_3_ concentration. The color at each position in the plotted map represents the mean intensity of the spectrum measured at that specific location. Each dried spot’s mean intensity values are normalized per spot to highlight intensity variations within each dried sample spot.

Figure 2 provides spatial information about the mean spectral intensity of each spectrum within the dried sample spot. Due to the heterogeneous intensity distribution, absolute sample concentrations cannot be directly derived from Raman intensity alone. However, Figure 1 suggests that the spectra contain information about protein content, using the adjuvant Al­(OH)_3_ as a potential calibration substance. To explore this, a nonblind unmixing approach was applied using non-negative least-squares (nnls), leveraging prior knowledge of the pure spectral components to estimate their contributions to each individual mixed spectrum. nnls uses the reference spectra shown in Figure 1B and calculates the contributions of BSA and Al­(OH)_3_ across the sample spot, from which the concentration ratios can be derived. The calculated concentration ratios correlate well with the known mixture composition and show a homogeneous spatial distribution (Figure 3). As the relative BSA concentration in the mixture increased from the leftmost to the rightmost spot in Figure 3, the estimated BSA:Al­(OH)_3_ concentration ratio also increased. This qualitative illustration demonstrates that, despite the heterogeneous spectral intensity observed in Figure 2, ratios of BSA to Al­(OH)_3_ remains spatially uniform across the sample spots up to a BSA concentration of 2 mg/mL. At a higher concentration ratio of 5 mg/mL, however, a pattern emerges: less BSA was detected in the inner spot compared to the outer ring. Here, the adsorption capacity of Al­(OH)_3_ was exceeded, as a portion of the BSA remained in the supernatant after centrifugation rather than being fully adsorbed (Figure S1).

**Figure 3:**
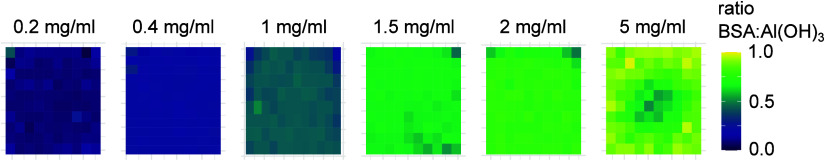
Variation in estimated concentration ratios of BSA to Al­(OH)_3_ across dried sample spots. Concentration ratio of BSA to Al­(OH)_3_ for each spectrum across the spots were estimated by computing coefficients with nnls as qualitative illustration. Coefficients for both BSA and Al­(OH)_3_ in the mixture were computed for each spectrum across all sample spots, based on their respective reference spectra. During the computational process, nnls iteratively adjusts the coefficients while enforcing non-negativity, ensuring that the reconstructed mixture spectrum remains a physically meaningful linear combination of the pure component spectra. Normalization was done across all spots with one overarching scale.

This observation supports the hypothesis that it will be possible to reliably extract information about protein concentrations relative to a internal standard (here Al­(OH)_3_ adjuvant) from the Raman spectral fingerprint of full vaccines.

### Spectral Unmixing: Identification and Quantification of the Protein Component

Raman spectra of the pure components (BSA and Al­(OH)_3_) are known in the sample mixtures of the current study, allowing the application of regression methods such as nnls to determine component contributions. This can be considered as a nonblind unmixing method, as the pure components are known a priori. Yet, this is not generally the case for more complex vaccine formulations with proteins adsorbed to adjuvants. In these cases, pure component spectra need to be estimated from the spectrum of the mixed sample employing computational unmixing methods (blind unmixing methods). In this study, the performance of an autoencoder neural network was evaluated as a hyperspectral unmixing method to identify individual components, known as endmembers, and their proportions in the mixtures.^22^ AE consists of two sequentially connected subnetworks: the encoder and the decoder. The encoder transforms the input data *x* into a lower-dimensional latent space representation, while the decoder reconstructs the original input *x̂*. The AE is trained by minimizing the difference between the input x and the reconstruction *x̂*, thereby capturing the most essential characteristics of the input spectra within the latent space. When the dimension of the latent space is defined as the number of expected components in the mixtures, the component proportions (abundances) can be interpreted from the latent space. In addition, individual pure component spectra can be obtained directly from the learned weight matrix of the decoder, where each column represents an endmember spectrum.

We compared the results of AE in extracting endmembers (pure component spectra) and their abundances to those obtained using the state-of-the-art unmixing method, Multivariate Curve Resolution Alternating Least Squares.^20^ MCR is a regression technique that iteratively resolves mixed signals into individual components and quantifies their proportions by decomposing a data matrix of measured spectra.

For both methods, AE and MCR, a training set was used as input to calculate the two endmembers of the mixtures. The training set includes 1000 Raman spectra with a balanced distribution among five different BSA-Al­(OH)_3_ mixtures with varying concentration ratios. For each concentration ratio, two biological replicates were measured, each consisting of 100 spectra.

Initial analysis showed that an AE trained on 1000 measured spectra identified the endmembers (Figure S3) with low signal-to-noise ratio. Since deep learning models such as autoencoder are known to require a large data set to reliably identify patterns in the data, we generated synthetic out-of-distribution data and incorporated them into the input for AE training.

### Benefits of Adding Synthetic Data to the Original Training Data Set of the AE

Incorporating synthetic spectra into the training set can improve the performance of an autoencoder in unmixing spectra of BSA-Al­(OH)_3_ mixtures into their individual pure component spectra (Figure 5). We utilized Contextual Out-of-Distribution Integration (CODI)^33^ to generate synthetic spectra. CODI uses the variability seen in a subset of spectra, known as the calibration set, and incorporates this variability into the training set to generate additional synthetic spectra as illustrated in Figure 4. The left subplot shows a principal component analysis (PCA) of Raman spectra for BSA-Al­(OH)_3_ mixtures with varying concentrations. The right subplot displays a PCA of the same spectra but with additional synthetic spectra incorporated into the data set via CODI. The synthetic data reflect the variability observed in the original spectra as they are drawn from a distribution estimated from the original data (Figure S9). Instead of applying extensive preprocessing, outlier spectra visible in the PCA remained in the data set to enable downstream predictive models to learn both signal features and noise patterns, thereby improving its robustness and generalization.

**Figure 4:**
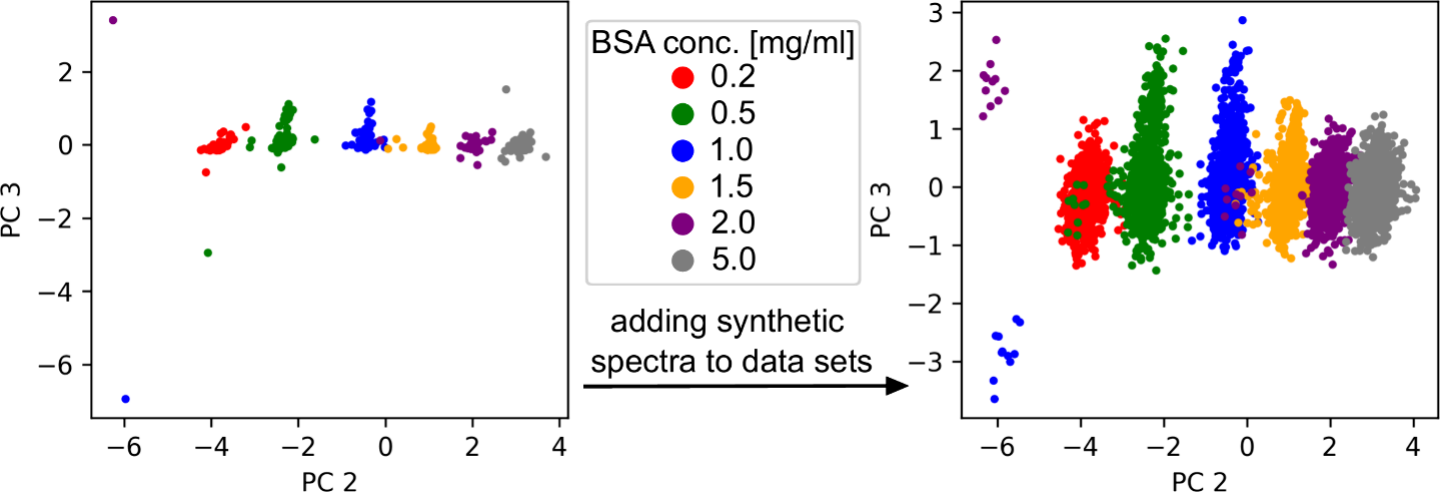
Effect of adding synthetic spectra to the data set. (Left) PCA score plot depicting Raman spectra for six BSA-Al­(OH)_3_ mixtures, each with different concentration ratios and 100 spectra per mixture. (Right) PCA score plot with additional synthetic spectra generated using CODI. Ten synthetic spectra were generated per measured spectrum by incorporating the variability observed within the 100 spectra of one measurement of a single concentration ratio into the measured spectra (within sample variability).

We evaluated the performance of AE models supplemented with different sets of synthetic data for training. The performance of each model is benchmarked by the cosine distance, as defined in eq 4, between the computed pure component endmember spectra and the reference spectra. The cosine distance measures the similarity between the extracted and measured pure component spectra; a lower cosine distance indicates greater similarity and thus better accuracy in endmember identification. Several sets of synthetic data were generated using different calibration data sets for the generation of synthetic spectra. Each calibration data set represents a different source of variability in the original data. These sources included: (1) the variability within a sample of 100 measured spectra (within-sample variability), (2) the variability seen among replicates of spectral measurements at the same concentration (within-concentration variability), (3) the variability seen among measurements at different concentration levels (between-concentration variability), (4) variability within the polystyrene calibration measurements conducted before measuring the samples of interest (within-calibration sample variability), or (5) no added synthetic spectra. The different calibration data sets were created as described in the Materials and Methods Section. For each spectrum in the original training data set, 15 synthetic spectra were generated according to the applied source of variability (1, 2, 3, or 4) and were added to the AE training data set. The size of the training set consequently increased from approximately 1000 Raman spectra of BSA-Al­(OH)_3_ mixtures with varying concentration ratios to approximately 15,000 spectra.

Autoencoders trained with additional synthetic data, irrespective of the variability source, outperformed the baseline autoencoder that was trained without synthetic data (Figure 5). The AE model that learned the synthetic data created from within-concentration variability slightly outperformed the other models in estimating the pure BSA component as well as slightly in estimating the concentration ratios (Figure S5). For further comparison against the performance of the MCR method, the AE model trained with synthetic data from within-concentration variability, was used. Our tests indicate that using multiple sources of variability in one training set does not enhance the unmixing performance compared to using a single source (Figure S4). The generation of between concentration variability might have led to some degree of obfuscation of the concentration dependent signal in the data. In a similar fashion, the introduction of variability seen in the calibration data set might not represent variability in actual data set as well as that covered by the within sample variability.

**Figure 5:**
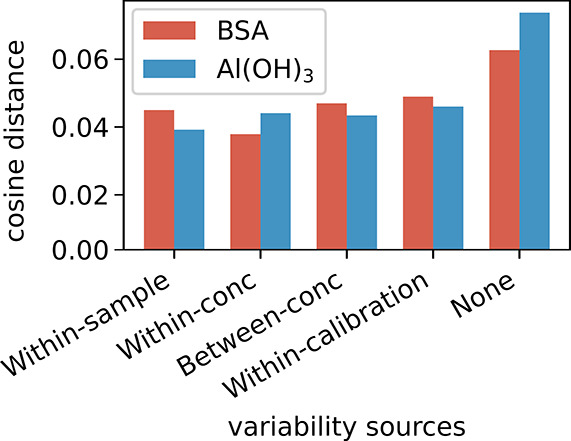
Benchmarking autoencoders trained with different sets of synthetic spectra. The AE models were trained on the original training data set supplemented by synthetic spectra generated using CODI with one of the following sources of variability: (1) within-sample variability, (2) within-concentration variability between technical replicates of the same concentration ratio, (3) between-concentration variability between samples with different concentration ratios, (4) within-calibration-sample of polystyrene measurements, or (5) no added synthetic spectra. Each bar represents the cosine distance between the pure component spectrum estimated by the model and the measured pure components spectrum, for BSA (red) and Al­(OH)_3_ (blue). Small cosine distance indicates greater similarity and better model performance.

The performance of the autoencoders used for endmember extraction is influenced by both the source of variability in the synthetic data and the number of synthetic spectra added to the training set (Figure 6). Several AEs were trained on data sets of varying sizes by incorporating different quantities of synthetic data each generated with different random seeds. As the number of synthetic spectra in the training set increased, the unmixing performance improved. The cosine distance between the computed pure component spectra and their references decreased, reaching a minimum with approximately 15,000 (10^4.2^) spectra in the training data set. The mean ICV also improved with larger training set sizes, reflecting the stability of the predicted endmember spectra across repeated predictions using five different synthetic data sets for each training set size. However, we observed that an excessive number of synthetic spectra led to a decline in performance (Figure 6), indicating that the true signal may be obscured by excessive synthetic data. Stable and successful predictions were achieved once the training set reached approximately 10^4.2^ synthetic spectra. Consequently, the results presented in the manuscript were generated using a fixed seed of 3 to ensure exact reproducibility. Fifteen synthetic spectra were generated for each original spectrum, resulting in a training set of 15,000 synthetic and 1000 original spectra.

**Figure 6:**
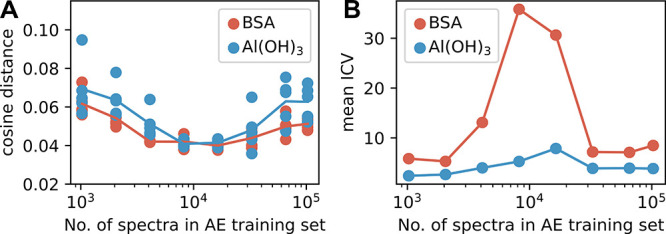
Performance of autoencoder in extracting pure component spectra (endmembers) relative to synthetic-augmented training set size. Synthetic spectra were generated via CODI to incorporate the within-concentration variability into the training set. Five different AE models were trained for any given number of spectra in the training set. The differences between the models originate from a degree of randomness in the synthetic data generation. (A) Cosine distance between extracted pure component spectra from the AE model and their reference reaches its minimum with approximately 15,000 (10^4.2^) spectra in the training set. (B) Increase in the inverse coefficient of variation (ICV) (eq 6) with larger training data sets up to approximately 10,000 before a decrease is observed. The *x*-axes show the numbers of spectra in log scale.

### Evaluation Unmixing Methods

#### Estimate of Pure Component Spectra: Identification of Components

To evaluate the performance of both methods (AE and MCR) in computing pure component spectra (endmembers) from spectra of mixtures, we visually compared the computed endmember spectra with the measured spectra of pure components (Figure 7). In addition, we calculated the cosine distance between them (Figure 8A) to provide a quantitative assessment and repeated the prediction 5 times using the same input data but different random seeds (3, 15, 22, 34, 42). Both methods successfully extracted the two endmembers, BSA and Al­(OH)_3_. However, MCR showed superior performance in accurately extracting both endmembers, with only negligible differences between predictions arising from different matrix initializations due to random seed variation. In contrast, AE exhibit greater variability, which can be attributed to both the stochastic nature of synthetic data generation and the inherent randomness of the AE itself.

**Figure 7:**
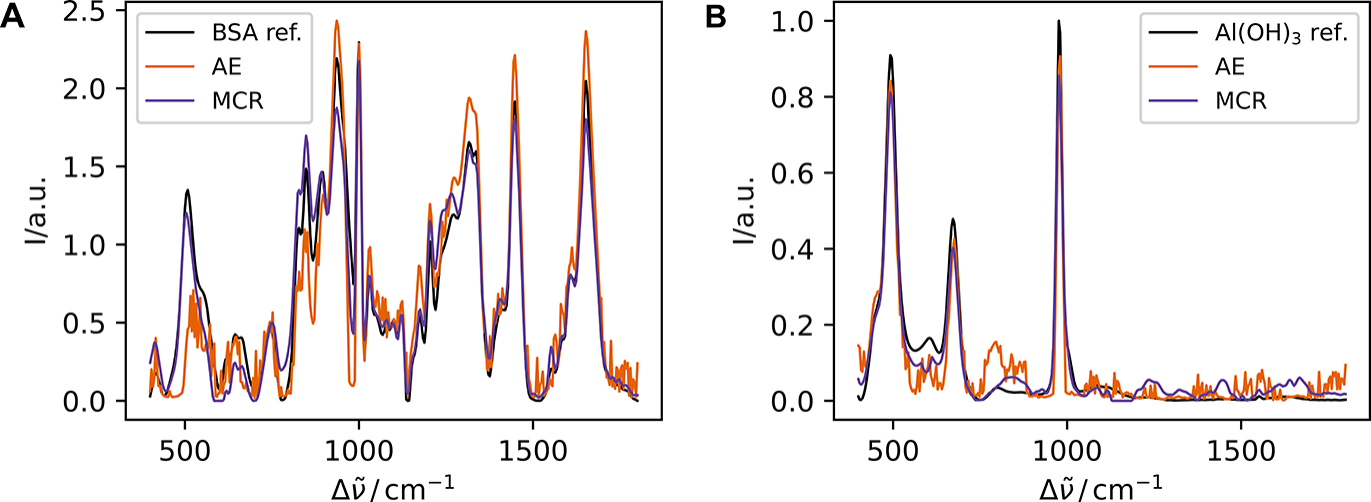
Comparative spectral analysis of estimated pure compounds. (A) Estimated spectra of BSA computed with MCR (purple) and with autoencoder (orange) alongside the reference spectrum of pure BSA (black). (B) Illustrates the same for Al­(OH)_3_. All spectra are area-normalized to account for the inherent scale ambiguity in the unmixing models, enabling a direct comparison of extracted vibrational signatures and peak ratios independent of absolute amplitudes. The autoencoder was trained on the original training data set supplemented by additional synthetic data generated using the CODI method, whereas the MCR results are based solely on spectra from the original training data set.

#### Estimate of Concentration Ratios: Quantification of Protein Content

Concentrations of BSA in relation to Al­(OH)_3_ (BSA:Al­(OH)_3_ concentration ratios) were estimated using autoencoder and multivariate curve resolution methods. These estimations were performed on spectra from mixtures that had been used for model training as well as on previously unseen test spectra. The trained AE model processed the mixture spectra, and the estimated concentration coefficients were extracted from the latent space. These concentration coefficients or ratios can be used to estimate the BSA concentrations in the original samples as Al­(OH)_3_ adjuvant concentration serves as an calibration substance which has consistently been set to 0.5 mg/mL. For the corresponding application of the MCR method, endmembers computed from the training data set using the MCR method were utilized, and the concentration coefficients for the mixtures were estimated using the non-negative least-squares principle, a fundamental component of the MCR approach. Three independent data sets were collected, with one serving as the training set and the remaining two as test data sets. The sample preparation protocols for the test sets differed slightly: in the second test data set, an additional washing step was implemented to ensure the removal of any residual unbound BSA. SDS-PAGE analysis of the pellet samples confirmed that no nonadsorbed BSA remained detectable in either preparation. As both protocols yielded comparable results, both test data sets were included in the subsequent analysis. The estimated concentration coefficients were benchmarked by evaluating the mean squared error (MSE) between the computed and true concentrations, averaged across all replicates in the training or test set (Figure S6A). The corresponding standard deviation was visualized with error bars; for the exact calculation, refer to eq 5. Figure S6A presents the test set predictions for each concentration (ratio), individually averaged across all four replicates, along their respective standard deviation. The results showed that the predictions of the AE model outperformed those of the MCR method in both the training and the test data sets. Both methods showed the expected underestimation of BSA concentration at the highest ratio of 5 mg/mL (Figure S6B), which is consistent with the known adsorption capacity limitations of 0.5 mg/mL Al­(OH)_3_ and the results from the SDS PAGE (Figure S1). Therefore, only the concentrations 0.2, 0.5, 1, 1.5, and 2 mg/mL BSA adsorbed to 0.5 mg/mL Al­(OH)_3_ were considered when computing the MSE. Figure 8B shows the estimated concentration of BSA in the original sample, as determined by AE and MCR, plotted against the expected concentration. Autoencoders consistently showed better estimates of concentration ratios on unseen spectral data than the MCR algorithm. Mean BSA concentrations (ranging from 0.2 to 2 mg/mL) and the corresponding relative standard deviations (RSDs) were calculated based on two replicates (dots 1 and 2) from two independent test sample preparations (data sets 1 and 2). The BSA concentrations determined using AE exhibited a linear correlation with the expected values, with RSDs between 1.2% and 11% (Table S1). Similarly, for the third test set (fixed BSA and varying Al­(OH)_3_), the AE-predicted values maintained precision with RSDs up to a maximum of 12.4% (Table S3). In comparison, BSA concentrations obtained via MCR resulted in RSDs ranging from 3.6% to 14.1% (Table S2).

## Discussion and Outlook

Identification and quantification of protein in multicomponent aqueous solution is a challenging task—yet a prominent requirement in the quality control of biomedicines such as vaccines. This is especially true for adsorbed and multivalent vaccines,^9^ where the presence of adjuvant adsorbed antigens complicates quantification using classical protein assays for determination of the protein concentration.^12^ We have evaluated the potential of Raman spectroscopy to determine protein concentration in a synthetic model vaccine in which BSA is adsorbed to Al­(OH)_3_. Raman spectroscopic measurement of air-dried sample spots requires careful consideration of spatial heterogeneity, but ensures sufficient signal intensity for protein identification. We have shown that protein content can be derived quantitatively in this setting as the spectral signal contains information about the ratio between protein and adjuvant concentrations in the sample. This shows the potential of Raman spectroscopy as an analytical technique for the characterization of protein-adjuvant formulations in relevant concentration ranges—despite the fact that proteins, in particular, adsorbed proteins—show a weak Raman signal as compared to those of typical excipients. This could be facilitated by using the spectral contribution of excipients such as aluminum hydroxide adjuvants as an internal standard, enhancing the reliability of relative protein quantification. Furthermore, spectral unmixing algorithms have proven effective in identifying the unique spectral fingerprints of pure components and detecting them within complex mixtures.

Computational unmixing of samples was specifically performed and benchmarked using a neural network autoencoder and multivariate curve resolution as an iterative regression technique. AE outperformed the MCR method on both training and test data sets (Figure S6A) in estimates of relative concentrations of components. However, it is important to note that while the autoencoder excelled in concentration estimation, MCR showed better performance in the identification of the endmembers (pure component spectra) (Figure 8A). This difference in performance could be attributed to the underlying assumptions of the methods. MCR, as well as the integrated concept of non-negative least-squares regression, assumes linearity in the spectral decomposition. However, as demonstrated in Figure S8, the measured intensity ratio between the BSA peak (1000 cm^–1^) and the Al­(OH)_3_ peak (976 cm^–1^) follows a nonlinear trend of both components after adsorption when compared to the actual concentration ratios in the sample. Because MCR is a linear model, the slight overestimation at lower concentrations and underestimation at higher concentrations (Figure 8B) are consistent with the onset of adsorption saturation observed in the two highest-concentration mixtures (Figure S1), where the fixed amount of Al­(OH)_3_ cannot adsorb all BSA, leaving an unmeasured fraction in the supernatant. Autoencoders offer more flexibility in modeling complex relationships, which results in better performance in estimating relative concentrations. In a similar fashion, this nonlinearity feature might be advantageous in the context of other advanced photonic technologies.^35–37^ The additional loss function guides the autoencoder toward latent representations that not only reconstruct the spectra but also reflect realistic concentration distributions. This suggests that AEs may be better suited to handle scenarios that are not constrained to linear mixing of spectral contributions. It is worth noting that other methods of hyperspectral decomposition like Vertex Component Analysis (VCA)^38^ and N-FINDR^39^ often struggle when pure component spectra are not present in the data set, as they rely on the geometric concept of pure component spectra residing at the vertices of a simplex. In contrast, AE and MCR demonstrate the ability to extrapolate the pure component spectra of mixtures even when the pure spectra are not explicitly contained within the analyzed data set. This is particularly relevant for adsorbed proteins, where obtaining a representative pure spectrum of the antigen in its adsorbed state is often not feasible due to conformational changes or matrix interactions.

**Figure 8:**
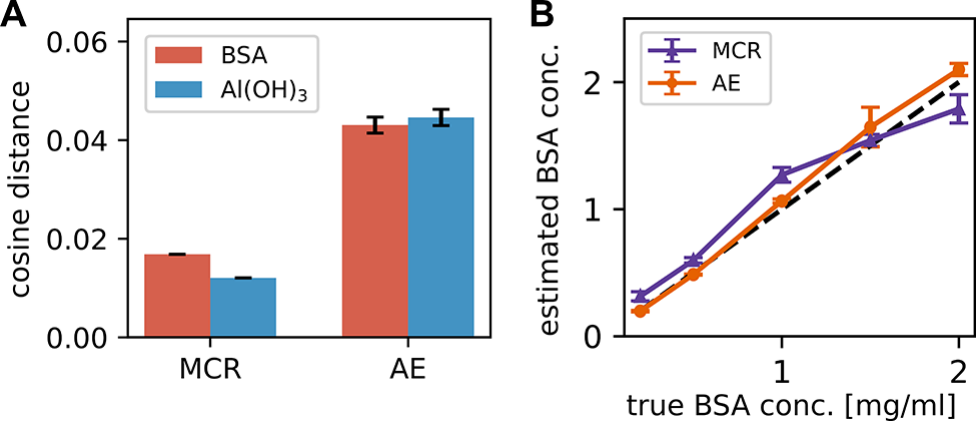
Unmixing performance comparison between MCR and AE. (A) Cosine distance between the computed endmember spectra and reference pure component spectra. Error bars represent the standard deviation (SD) across five independent runs. For the AE, variability originates from different random seeds used for data augmentation; for MCR, variability stems from different random matrix initializations. (B) Predicted vs true BSA concentrations. Solid lines represent the mean predictions across four replicates (two technical replicates from two independent test sets), with error bars indicating the SD (*n* = 4). AE predictions demonstrate superior alignment with the expected concentration values compared to the MCR method (Figure S6).

Another technical contribution lies in the integration of CODI-based (Contextual Out-of-Distribution Integration) data augmentation within the spectral unmixing workflow. This improved the model’s robustness and generalization to unseen data with similar variability. The synthetic data expanded the original training set, incorporating underrepresented sources of variance and enabling the autoencoder to learn a more informed decision boundary for unmixing BSA-Al­(OH)_3_ mixtures. The choice of variability source was critical to performance and the AE utilizing within-concentration variability was chosen as it shows highest accuracy in both pure protein identification and concentration estimation compared to models trained solely on limited experimental data (Figure 5, Figure S5). This likely arises because they capture the inherent heterogeneity present within individual sample measurements of the same product due to factors such as instrument noise and local sample inconsistencies, which are crucial aspects of real-world data. However, excessive interclass variance can lead to latent space overlap, which hinders the model’s ability to distinguish between distinct concentration levels. The observed decline in performance at high augmentation levels, at ≈10^4^ synthetic spectra and above (Figure 6), indicates that excessive synthetic data begins to dominate the training set, effectively diluting the real spectra signal. CODI is more suitable than arbitrarily adding noise because it leverages empirically observed variability from calibration data sets, allowing the synthetic data to more accurately reflect the real-world variations present in the measurements, leading to better model generalization.^33^ CODI’s use of mean-centered differences in its calibration data set ensures that the introduced variability is relative to the average signal, thus preserving the overall spectral characteristics while adding context-aware perturbations to the seed data. Synthetic data were only considered for the autoencoder training as MCR’s performance can degrade with a larger number of spectra due to the increased complexity of the underlying linear decomposition.

In this study advances could be demonstrated in combining Raman spectroscopy with machine learning methods for the characterization of complex biological samples. Yet for an application in quality control it is necessary to critically assess whether the obtained results meet the stringent requirements of modern pharmaceutical quality control. Particularly in protein quantification, both accuracy and precision are essential performance parameters. Accuracy requirements (recovery expressed as mean concentration relative to expected concentration) typically range from 90% to 110% for biophysical methods or from 80% to 120% for biological methods. In this study, BSA concentrations in Al­(OH)_3_ adsorbed samples were quantified using a model based on AE. The model yielded recovery rates ranging from 97% to 110% at different concentration levels (Table S1). Furthermore, the additional third test set, involving varying adjuvant concentrations, demonstrated recovery rates between 80% and 115% (Table S3). Acceptable limits for precision of biological methods (expressed as relative standard deviation, RSD) typically range between 5%–20% which were met by both methods (Table S1 and Table S3).

The approach offers a promising proof-of-concept, however further optimization and standardization is required to meet the required accuracy standards for routine quality control applications at the state of the art. It should be noted that this study utilized a relatively simple two-component model system with moderate spectral overlap to provide a foundational validation of the AE-CODI framework. Improvements of the method can be expected by addressing the following aspects: Limited variability in the training data has potentially limited the model’s robustness in handling naturally occurring sample variability. This includes the lack of temporal and procedural variation in the training set: The model was not extensively exposed to real-world variability introduced by preparing data sets across different days or conditions, which likely limits its generalizability. Any inconsistencies in handling, processing, or sample composition may introduce systematic or random errors. Although synthetic data augmentation improved prediction performance, it may also have reinforced some of these systematic biases (e.g., under- or overestimation at specific concentration ranges) due to replication of similar patterns within the augmented data set. To mitigate this, an extension of the data basis and application of orthogonal analytical methods needs to be applied to cross-validate results, identify potential biases, and enhance overall method robustness. However, this is out of the scope of the present study. In the future, we will expand the model system presented in this study by successively adding additional components, aiming to demonstrate that individual proteins can also be identified within more complex mixtures exhibiting three or more components. To identify potential biases and enhance overall methodological robustness, we will extend the underlying data basis by incorporating additional sources of variation, including different batches and sources of raw material. Furthermore, we will apply orthogonal analytical methods, including established quantitative reference techniques such as the Kjeldahl method, to cross-validate our machine learning based results. This will advance the method and take it a step closer to application in the regulatory context.

In conclusion, BSA concentration prediction is possible at typically required precision, improvement of the model and the training data is required to enhance predictive accuracy. Incorporating a broader range of real-world experimental data spanning different days, batches, and preparation conditions will likely strengthen the model’s capacity. This study highlights the potential of Raman spectroscopy combined with advanced computational methods to both identify individual components within complex biopharmaceutical formulations and estimate their concentration ratios based on full-spectrum measurements. Building upon the insights gained from this model vaccine system, future research will focus on improving and validating the underlying models as well as cross-validating the models with established quantitative reference techniques to enable their application in quality control settings. In the long term, these validated models may be extended to more complex formulations, such as tetanus or multivalent DTaP (Diphtheria, Tetanus, and Pertussis) vaccines. This will require the characterization of multiple antigens with distinct physicochemical properties adsorbed onto aluminum-based adjuvants. Preliminary data suggest that a differential identification and quantification of multiple proteins will be possible which would address current challenges in the characterization of DTaP vaccines. A systematic evaluation of Raman spectroscopy’s ability to resolve and quantify each component could eventually qualify the method as an alternative to animal experiments following a consistency approach. Ultimately, these advancements may facilitate the adoption of alternative or complementary analytical methods for the quality control of adsorbed vaccines.

## Supplementary Material



## Notes

Additional experimental details on SDS-PAGE including photographs and figures showing extended results (PDF)
