# Supplementary material for: Quantifying Components in a Model Vaccine with Machine-Learning-Augmented Raman Spectroscopy
Source: Anal Chem. 2026 Apr 28;98(18):13236–46. doi: 10.1021/acs.analchem.5c05538 (PMC13178555; doi:10.1021/acs.analchem.5c05538)
Supplement: Supplementary file 1 [file ac5c05538_si_001.pdf]

Supporting Information

Quantifying Components in a Model Vaccine with Machine-Learning-Augmented Raman Spectroscopy

Jana Hahn, [1,2,*] Pooja Gune,[1] Sascha Hein,[1] Wolf Holtkamp, [1] Marcel H. Schulz,[3] Walter Matheis,[4] Volker Öppling,[4] and Christel Kamp[1,2]

[1] *Paul-Ehrlich-Institut, Allergology Division, Central Method Development Section, 63225 Langen, Germany*

[2] *Bioinformatics, Goethe University Frankfurt, 60325 Frankfurt am Main, Germany*

[3] *Institute for Computational Genomic Medicine, Goethe University Frankfurt, 60590 Frankfurt am Main, Germany*

[4] *Paul-Ehrlich-Institut, Infectious Diseases Division, Quality Assessment Vaccines Section, 63225 Langen, Germany*

[5] *Paul-Ehrlich-Institut, Infectious Diseases Division, Product Testing Vaccines Section, 63225 Langen, Germany*

[*] *Corresponding Author Email: jana.hahn@pei.de*

List of Figures

S1 **SDS-PAGE** S2

S2 **Variation in spectral intensities across dried sample spots of references** S3

S3 **Comparative spectral analysis of estimated pure compounds without synthetic spectra in the training set** S4

S4 **Benchmarking of additional AEs that were trained with different sets of synthetic spectra: Endmember evaluation** S4

S5 **Benchmarking AEs that were trained with different sets of synthetic spectra: Concentration ratios evaluation** S5

S6 **Unmixing performance and concentration estimations ratios** S5

S7 **Concentration ratios of replicates in test set 3** S7

S8 **Intensity ratio of BSA and $Al(OH)_3$ peaks across known mixtures** . S7

S9 **Spectral Fidelity Analysis** S8

List of Tables

S1 Autoencoder predicted BSA concentration [mg/mL] in mixtures. S6

S2 MCR predicted BSA concentration [mg/mL] in mixtures. S6

S3 AE predicted concentration ratios of test set 3 . . . . S6

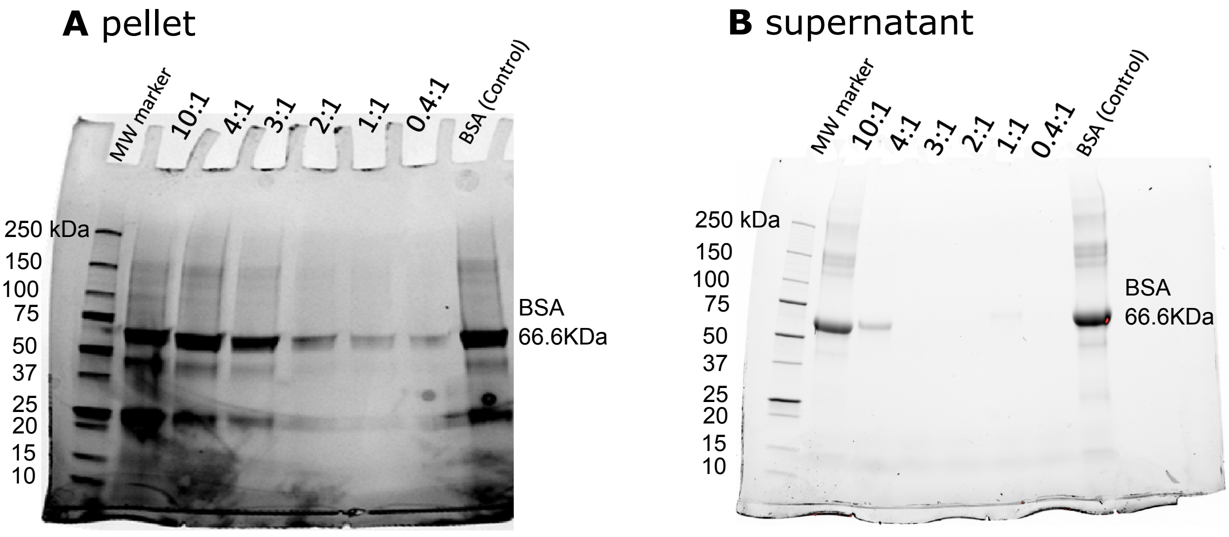

Figure S1: SDS-PAGE
Bovine Serum Albumin (BSA) at concentrations of 5, 2, 1.5, 1, 0.5, 0.2 mg/mL was mixed with 0.5 mg/mL of Aluminum hydroxide adjuvant suspension at equal volumes for adsorption. The mixture can be separated into two distinct phases: the pellet and supernatant. The presence of protein in the pellet as well as the supernatants were accessed by SDS-PAGE. Pure BSA was added as a control for reference bands. Panel A shows the increasing protein concentration in the pellet with increasing protein to adjuvant ratios. Panel B shows the emergence of protein in the supernatant beginning at concentration ratios of 4:1 indicating the onset of saturation in protein adsorption.

SDS PAGE (Sodium Dodecyl Sulfate Polyacrylamide Gel Electrophoresis) was used to

analyze pellets and supernatants recovered from the adsorption experiment. The samples were separated by molecular weight using 4-20% Mini Protean TGX (Tris Glycine eXtended shelf life) stain-free precast protein gels. 15 µl of each sample was mixed with 5 µl of Roti load1 (reduced), a 4x concentration dye. The sample mixtures then heated at 80 Celsius for 10 minutes, then placed on ice for 5 minutes. The SDS-PAGE chamber was prepared by inserting ready-to-use Mini protein TGX stain free protein gel cassettes into the electrode unit. An unstained molecular weight standard (10 - 250 KDa) was also loaded into gel. Protein samples were then loaded and electrophoresed at 140 V for 45 minutes using 1x SDS running buffer. The image of the separated protein bands was captured using the Fusion FX Imager under UV illumination with an ethidium bromide (EtBr) filter (F 590). The imager activates the reaction between the proteins and trihalo compounds in the gel, enabling visualization of the bands.

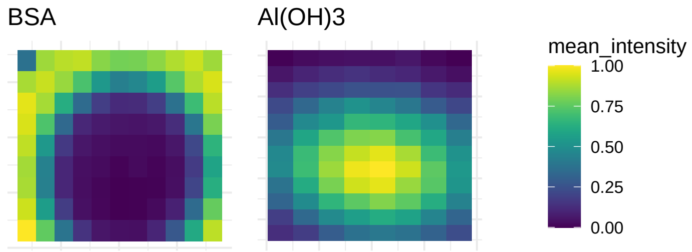

Figure S2: Variation in spectral intensities across dried sample spots of references Colormaps of mean spectral intensities across dired spots of pure BSA and pure $Al(OH)_3$. Each position on the map represents a spectrum that was measured at this position in the dot. The colorscale was normalized across each dot and indicates the mean spectral intensity.

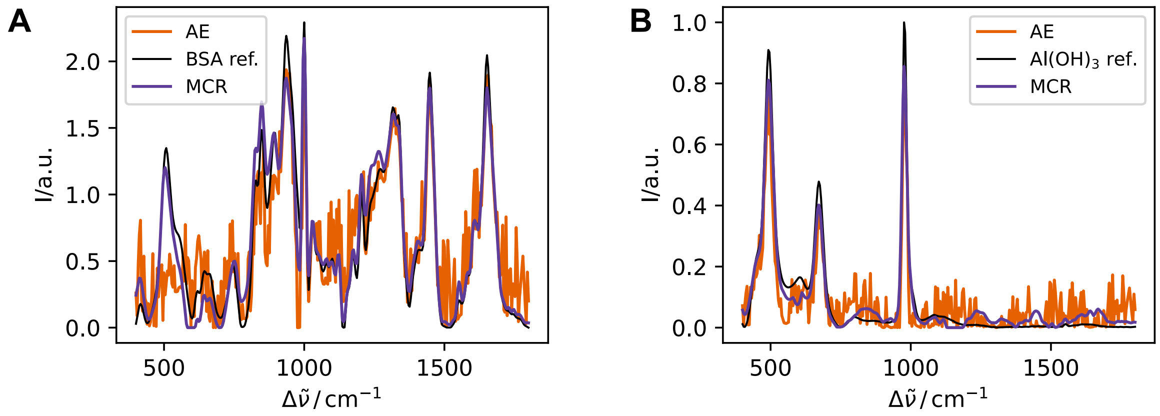

Figure S3: Comparative spectral analysis of estimated pure compounds without synthetic spectra in the training set
A: Estimated spectra of BSA computed with MCR (purple) and with autoencoder (orange) alongside the reference spectrum of pure BSA (black). **B**: Illustrates the same for $Al(OH)_3$. Both, autoencoder and MCR computed the endmembers from the original training data set not supplemented by additional synthetic data. All spectra are area-normalized to account for the inherent scale ambiguity in the unmixing models.

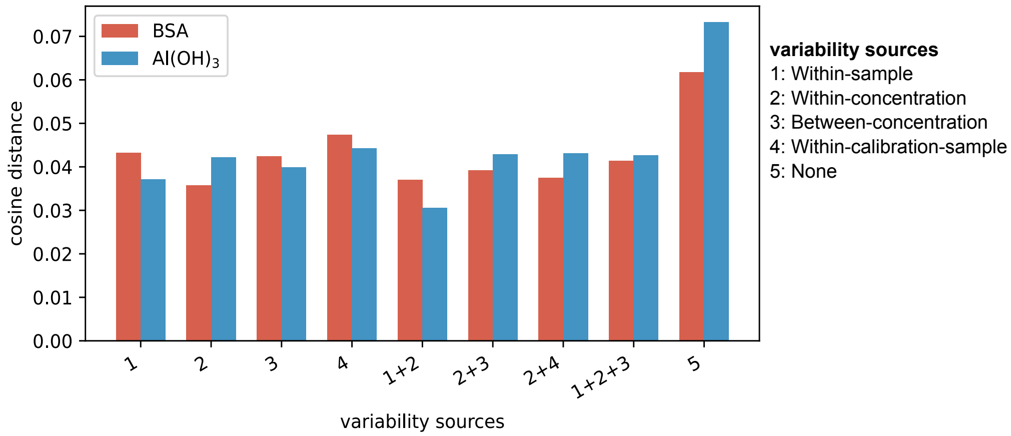

Figure S4: Benchmarking of additional AEs that were trained with different sets of synthetic spectra: Endmember evaluation
Several sets of synthetic spectra were generated using different sources of variation as well as a combination of different sources. Each bar represents the cosine distance between the estimated and measured pure components BSA (red) and $Al(OH)_3$ (blue). The autoencoder were trained on additional synthetic spectra that were generated with CODI based on the following variability sources: 1. within sample variation, 2. Within concentration variation, 3. Polystyrene calibration , 4. no added synthetic spectra, including a selection of combinations. These combinations were selected because they seemed most promising in improving the endmember prediction. However a combination of several variability sources did not improve the results compared to using a single variability source for synthetic data generation via CODI.

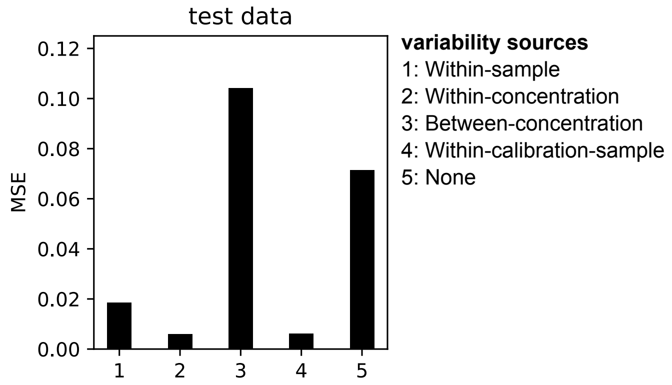

Figure S5: Benchmarking AEs that were trained with different sets of synthetic spectra: Concentration ratios evaluation
Mean squared error (MSE) between the estimated and true concentration ratios (0.2, 0.5, 1, 1.5, and 2 mg/mL BSA to 0.5 mg/mL Al(OH)3) as described in Eq. 5. Each bar represents the mean-squared error of the estimated concentration ratios of the four different AEs. Generating synthetic spectra via CODI and adding them to the data set for training improve the concentration ratio prediction of the AE model (cf. AE model 1, 2, 3 or 4). Autoencoders trained with synthetic data, aside from variability source 3, surpassed the baseline model (5). The AE using within-concentration (2) and within-calibration (4) variability performed best in estimating component concentration ratios. The AE incorporating between-concentration variability (3) underperformed relative to the baseline (5). This suggests that the synthetic data introduced excessive variance between the concentration classes, leading to an overlap in the latent space that hindered the model's ability to distinguish distinct concentrations.

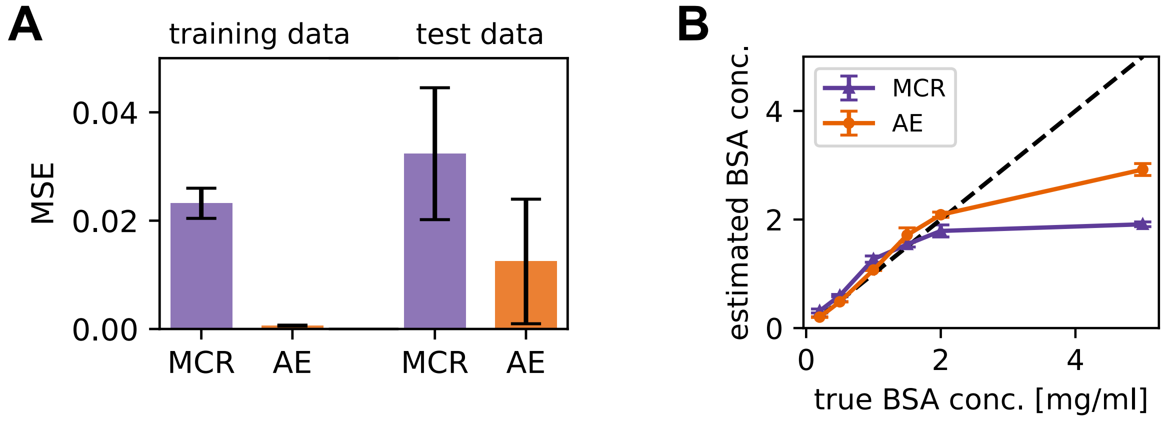

Figure S6: Unmixing performance and concentration estimations ratios
A: Mean squared error (MSE) (Eq. 5) between the estimated and true BSA concentrations in the mixtures (0.2, 0.5, 1, 1.5, and 2 mg/mL BSA to 0.5 mg/mL $Al(OH)_3$). The plotted MSE was averaged across these five BSA concentrations. Error bars represent the variation of predictions on different subsets of replicates in the training set as well as in the two test sets (each containing duplicates). **B**: Estimated vs. true concentrations including highest BSA concentration of 5mg/mL.

Both methods MCR and AE were used to predict the concentration ratios of the two compounds BSA and $Al(OH)_3$ in the mixtures. To conclude about the actual BSA concentration, the known and constant $Al(OH)_3$ (0.5 mg/mL) concentration after mixing 1:1 was used to conclude about the actual BSA concentration in the samples.

Table S1: Autoencoder predicted BSA concentration [mg/mL] in mixtures.

True BSA	Training Set		Test Set 1		Test Set 2		Mean	RSD%	Recovery%
**conc.**	**dot1**	**dot2**	**dot1**	**dot2**	**dot1**	**dot2**	**test data**	**test data**	**test data**
0.2	0.19	0.19	0.19	0.19	0.20	0.21	0.20	4.85	98.8
0.5	0.49	0.49	0.48	0.49	0.49	0.48	0.49	1.19	97.0
1	1.02	0.99	1.06	1.04	1.09	1.07	1.07	1.95	106.5
1.5	1.52	1.54	1.49	1.49	1.83	1.77	1.65	10.98	109.7
2	1.95	1.97	2.04	2.11	2.17	2.07	2.10	2.68	104.9

Table S2: MCR predicted BSA concentration [mg/mL] in mixtures.

True BSA	Training Set		Test Set 1		Test Set 2		Mean	RSD%	Recovery%
**conc.**	**dot1**	**dot2**	**dot1**	**dot2**	**dot1**	**dot2**	**test data**	**test data**	**test data**
0.2	0.19	0.20	0.33	0.33	0.25	0.35	0.32	14.09	157.5
0.5	0.56	0.59	0.57	0.60	0.60	0.63	0.60	4.08	120
1	1.01	1.15	1.27	1.36	1.20	1.25	1.27	5.26	127
1.5	1.45	1.44	1.51	1.51	1.51	1.62	1.54	3.58	102.5
2	1.70	1.70	1.61	1.84	1.81	1.90	1.79	7.02	89.5

Table S3: AE predicted concentration ratios of test set 3
Results for mixtures with a fixed $Al(OH)_3$ concentration (0.5 mg/mL) and varying BSA concentrations (0.2, 0.5, 1, 1.5, 2, and 5 mg/mL). Each concentration was measured in duplicate (technical replicates).

True conc.	Test Set 3				
**ratio**	**dot 1**	**dot 2**	**Mean**	**RSD%**	**Recovery%**
0.25	0.19	0.20	0.20	5.0	80.0
0.34	0.37	0.36	0.37	2.7	108.8
0.50	0.50	0.50	0.50	0.0	100.0
1.00	1.15	1.14	1.15	0.9	115.0
2.50	2.86	2.40	2.63	12.4	105.2

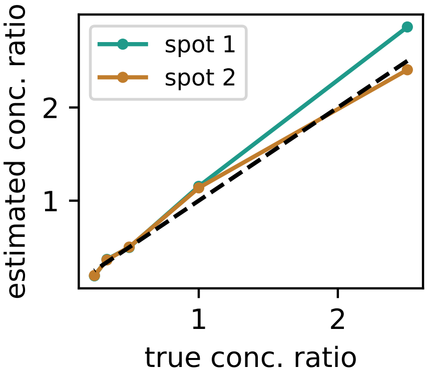

Figure S7: Concentration ratios of replicates in test set 3
Test set 3 contains samples with a fixed BSA concentration and varying $Al(OH)_3$ concentrations

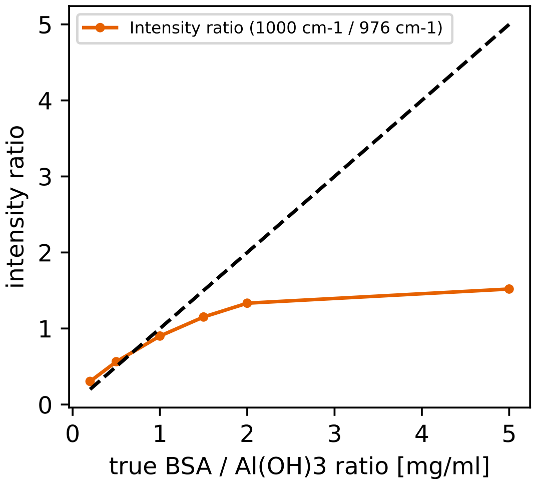

Figure S8: Intensity ratio of BSA and $Al(OH)_3$ peaks across known mixtures
Measured intensity ratio between the BSA peak at 1000 cm^{-1} and the $Al(OH)_3$ peak at 976 cm^{-1} plotted against true BSA/$Al(OH)_3$ concentration ratios. The experimental data show a non-linear trend.

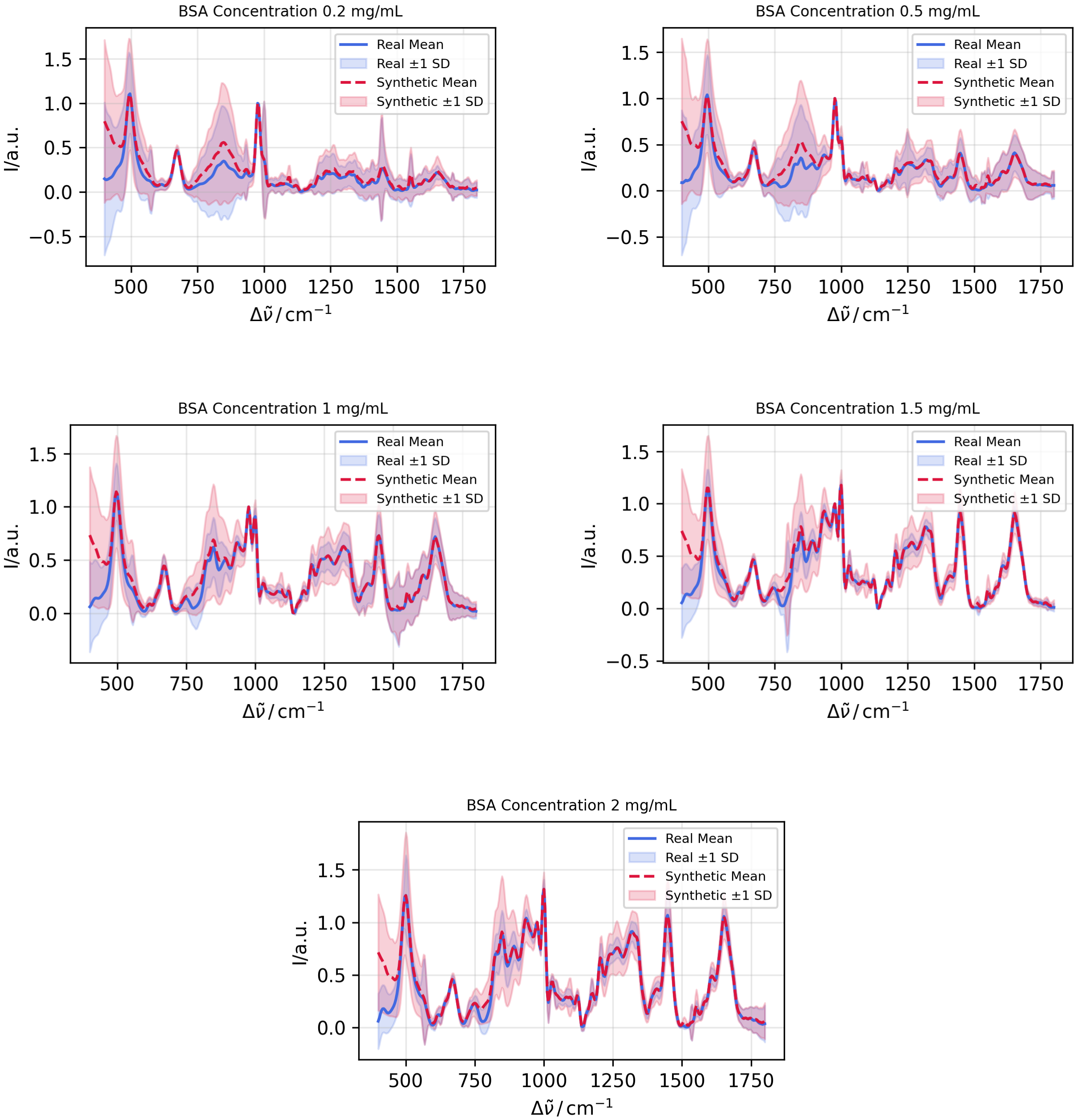

Figure S9: Spectral Fidelity Analysis
Comparison of real and synthetic Raman spectra across varying BSA concentrations. Shaded areas represent ±1 standard deviation. The synthetic spectra accurately replicate the characteristic peak positions, relative intensities, and experimental variance (standard deviation) across the primary regions.
